# Experimental Validation of the Cementation Mechanism of Wood Pellet Fly Ash Blended Binder in Weathered Granite Soil

**DOI:** 10.3390/ma16196543

**Published:** 2023-10-03

**Authors:** Jebie Balagosa, Min-Jy Lee, Yun-Wook Choo, Ha-Seog Kim, Jin-Man Kim

**Affiliations:** 1 Department of Civil and Environmental Engineering, Kongju National University, 1223-24, Cheonan-daero, Seobuk-gu, Cheonan-si 31080, Republic of Korea; jebie.balagosa@gmail.com (J.B.); leemg1215@naver.com (M.-J.L.); 2 Department of Architectural and Engineering, Kongju National University, 1223-24, Cheonan-daero, Seobuk-gu, Cheonan-si 31080, Republic of Korea; bravo3po@kongju.ac.kr (H.-S.K.); jmkim@kongju.ac.kr (J.-M.K.)

**Keywords:** sustainable construction material, wood pellet fly ash blended binder, unconfined compressive strength, suction tests, microstructural analyses

## Abstract

In response to climate change, wood pellets have been increasingly utilized as a sustainable energy source. However, their growing utilization increases the production of wood pellet fly ash (WA) by-products, necessitating alternative recycling technologies due to a shortage of discharging landfills. Thus, this research seeks to utilize WA by developing a new sustainable construction material, called wood pellet fly ash blended binder (WABB), and to validate its stabilizing performance in natural soils, namely weathered granite soil (WS). WABB is made from 50% WA, 30% ground granulated blast-furnace slag (GGBS), and 20% cement by dry mass. WS was mixed with 5%, 15%, and 25% WABB and was tested for a series of unconfined compressive strength (*q_u_*), pH, and suction tests at 3, 7, 14, and 28 days. For the microstructural analyses, XRD, SEM, and EDS were employed. As the WABB dosage rate increased, the average *q_u_* increased by 1.88 to 11.77, which was higher than that of compacted WS without any binder. Newly cementitious minerals were also confirmed. These results suggest that the effects of the combined hydration mechanism of WABB are due to cement’s role in facilitating early strength development, GGBS’s latent hydraulic properties, and WA’s capacity to stimulate the alkaline components of WABB and soil grains. Thus, this research validates a new sustainable binder, WABB, as a potential alternative to conventional soil stabilizers.

## 1. Introduction

Soil is commonly stabilized by mixing soil, water, and chemical stabilizers, such as cement, lime, or other chemical additives, to strengthen soft and collapsible ground [1,2]. These stabilizers have been proven to be effective but may have potential environmental drawbacks such as carbon emission issues and economic considerations due to their associated production costs [3,4,5]. Specifically, cement manufacturing alone accounts for 5 to 8% of worldwide carbon emissions and 10% of global industrial energy consumption [6,7]. In recent years, there has been a growth in the popularity of alternative composite and eco-friendly binders, such as concrete applications [8,9,10] or soil stabilizers [11,12,13]. This can be attributed to their sustainability, cost-effectiveness, and distinctive chemical composition. Hence, the development of these construction materials represents a new frontier in the scientific quest to reduce the environmental impact of construction.

The biomass ash types previously explored as soil stabilizers include discharged waste from power plants [14,15,16] and industrial by-products [12,17,18,19]. Studies suggest that the stabilizing effect of biomass ash depends on the parameters of the soil mixture (i.e., soil type, the unique chemical and mineral content of the binder, and the water-to-binder ratio), the specimen preparation, and the curing conditions [13]. These biomass ashes contain some silica and alumina, exhibiting a soil-binding potential even when combined with moisture only [16,18,20]. However, the contribution of biomass ash to the mechanical properties of stabilized soil varies [21]. The combination of rice husk ash [22] and sawdust ash with clay [23] and wood ash-treated sand [16] results in positive enhancements in the mechanical properties. In contrast, olive waste ash-stabilized marl soil [24] and wood ash-stabilized clay [18] were observed to have limited efficacy due to their short-lived strength and low cementation capacity. These findings suggest that the strength gain of biomass ash-stabilized soil depends on the host soil, its unique chemical composition, and its capacity to produce cementitious minerals [13,21].

Despite the differences in the stabilization efficiency of biomass ash, biomass fly ash (BFA) is a good candidate for alternative cementitious materials due to its distinct strength gain when used as an admixture and due to its positive environmental and economic advantages [13,25]. One type of BFA that is largely understudied is wood pellet fly ash (WA) [26]. WA is a by-product of wood-pellet-based energy production. The use of wood pellets conforms with international trends regarding the use of biomass ash as a sustainable material for household and commercial heating fuel following ISO standards [27] and as one of the responses to the global problem of climate change. In particular, the South Korean government has developed an energy policy [28,29,30] to lessen the carbon emission issues associated with traditional fossil fuels [31,32]. As a result, the domestic demand for wood pellets is anticipated to increase [33]. This transition to the use of wood pellets in domestic power plants may further lead to a surge in its by-product, WA. The generated ash by-products make up roughly 2% of the fuel’s weight, and since 2014, over 350,000 tons of wood pellet fly ash have been produced annually, leading to landfill saturation [26]. Therefore, the excessive disposal of wood pellet fly ash in landfills has compelled researchers to develop recycling solutions.

However, despite the considerable presence of WA, there has been no significant advancement in the development of recycling technologies. Furthermore, in light of WA’s potential as a BFA, it is worth noting that there is currently a lack of research investigating WA’s effectiveness in soil stabilization. Additionally, the cementation mechanism of pure and blended BFA-stabilized albite-based weathered granite soil (WS) has rarely been analyzed. Thus, a unique challenge that this study aims to address is the excess production of WA by-products; the study deals with this issue by developing a wood pellet fly ash blend with low cement proportions while ensuring that the desired soil strength is achieved. 

## 2. Previous Work

Kim et al. [34,35,36] previously investigated the physical and chemical properties of raw and hardened wood pellet fly ash and the physical, chemical, and strength properties of WA ground granulated blast-furnace slag (GGBS)–cement blends for concrete and mortar applications. Their research findings show that raw wood pellet fly ash contains alkaline components, including K_2_O, Na_2_O, and MgO. In addition, a 27.8% CaO content was detected, suggesting its binding potential, especially for binders with 20% CaO (or greater) [18,37]. Interestingly, SO_3_, which could promote a high reactivity with Ca-based compounds [34,35], was identified; K_2_O, which is also beneficial to the strength gain [38] and alkali aggregate reactions, was also identified [34,35,36]. Moreover, raw WA contains low peaks of quartz and lime minerals; however, when WA is mixed with water, the hardened material shows a low peak of new hydrocalumite hydrate (based on XRD analysis). These findings imply that WA has unique hydraulic properties due to mineral changes during its production; the minimal strength contribution from WA’s hydration is due to its low hydrocalumite peak intensity.

In addition, a parallel study by Balagosa et al. [39] was conducted to investigate the wood pellet fly ash binding effect (5% to 25% by dry mass of soil) on weathered granite soil (WS) through dry mixing. The findings of the unconfined compressive tests [40] show that the measured strength gain of the WA-treated WS suggests premature and slow cementation for specimens with a low WA dosage (5% and 15% WA contents). Also, the slight 28-day strength decrease for 15% and 25% WA contents implies low WA hydration capacity when stabilizing WS [34,35,36,39]. Nevertheless, these findings motivated us to accelerate WA’s binding capacity; thus, we developed a sustainable construction material by creating a WA–GGBS–cement blend (predominantly made up of WA) and determining the optimal binder ratio through a series of experimental investigations. Firstly, cement activation tests were conducted on the WA–GGBS–cement blends (fresh and cured conditions) with a mixing ratio of WA (0% to 50%), cement (0% to 50%), GGBS (0% to 50%), and standard sand at a 1:3 ratio (binder-to-sand) and a water-to-binder (W/B) ratio of 50%; the blend was mixed to a slurry state in accordance with Korean Industrial Standards KS L ISO 679 [41] for cement strength test methods through air curing conditions for 3, 7, and 28 days. 

In the previous studies [34,35,36], freshly blended binder specimens were evaluated through slump flow (KS F 2594 [42]), setting time (KS F 2436 [43]), and hydration temperature testing methods. On the other hand, a series of compression tests (KS L 5105 [44]) and flexural strength tests (KS F 2408 [45]) were conducted on the cured specimens. The experiment results of the freshly blended binders show that the hydration of WA–GGBS and WA–cement is lower than that of the specimens mixed with pure cement and GGBS–cement blends. These findings suggest that WA is more suitable as an alkali stimulant than a hydration contributor. Interestingly, the cured specimens with 50% WA, 30% GGBS, and 20% cement were determined as the optimal design mix in the WA–GGBS–cement blend due to high unconfined compressive strength (*q_u_*) and flexural strength. Notably, the use of optimal *q_u_* is typically adopted in characterizing soil improvements and as a design parameter basis for ground improvement projects [46]. Thus, the present study adopted the 50% WA, 30% GGBS, and 20% cement ratio as a new sustainable soil stabilizer, called wood pellet fly ash blended binder (WABB), and extended its workability in natural soils in the field. It has been determined that the WA’s reactive properties are advantageous as an alkali stimulant, and the latent hydraulic characteristic of GGBS complements the strength gain in low-cement-based binders for concrete and mortar applications [34,35,36]. However, comprehensive analyses of the WABB cementation mechanism on albite-based weathered granite soil and natural field soils are lacking.

## 3. Objectives of This Study

This study aims to perform an experimental validation of the cementation mechanism of WABB in albite-based weathered granite soil. Specifically, this study investigated the following: (1) the influence of the WABB cementation mechanism (at 5%, 15%, and 25% dosage rates) on the mechanical properties of WS in unsaturated conditions; (2) the WABB’s efficiency in producing cementitious minerals in the microstructure of albite-based WS over a 28-day curing period, and (3) the WABB’s contribution to alkali ions and latent hydraulic characteristics, which are beneficial for ground improvement projects involving compacted WS. 

## 4. Materials and Methods

The WS used in this study came from a quarry in Cheonan, South Korea, with a median particle size (D_50_) of 1.21 mm [47], designated as Material Type 2 by AASHTO T294-92 [48] and classified as poorly graded sand (SP) by the Unified Soil Classification System (USCS). The WABB’s constituents are cement, GGBS, and wood pellet fly ash. The WA used in this study was discharged by Yeongdong Eco Power Plants, operated by Korea South-East Power Co., Ltd., (KOEN), wherein a hybrid dust electric precipitator at the top of the boiler gathers the by-products created during the pyrolysis of wood pellets for energy consumption. WA often has a dark gray color, a dry density of 2.31 g/cm^3^, a fineness (measured using the Blaine method) of 3350 cm^2^/g, and a pH of 12 or higher. The physical attribute of WA is characterized by its unique fine particles with a median size (*d_50_*) of 15.347 × 10^−3^ mm. BFA, with a particle size ranging from 0.004 to 0.1 mm [16,49,50,51], is a promising candidate as a soil stabilizer for the following reasons: BFA has a small particle size capable of effective strength enhancement when used in mortar applications [52,53] and as a soil stabilizer [21,54]. In relation to this, there may be lesser production costs when using BFA as a soil stabilizer since the pre-processing of coarse raw materials is required to increase binding efficiency [53,55]. Thus, this evidence suggests that WA is a promising sustainable construction material.

**Figure 1:**
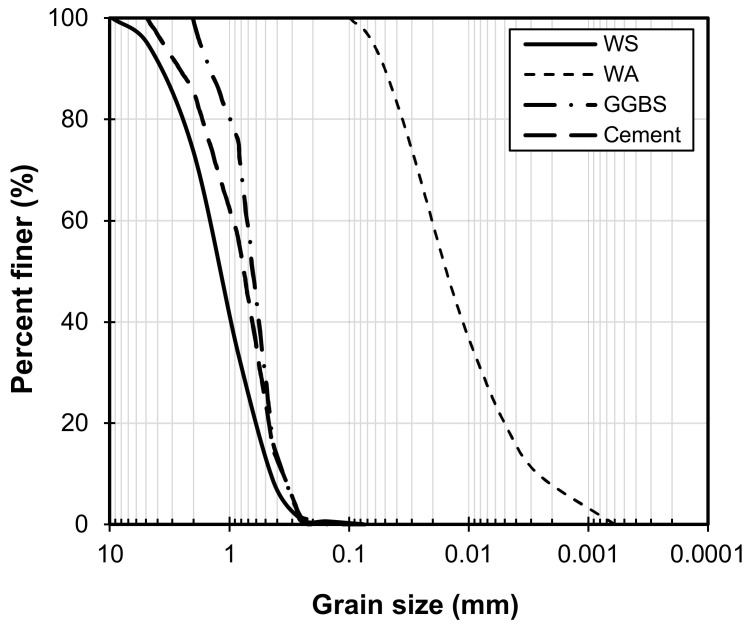
Particle size distributions of weathered granite soil, ground granulated blast-furnace slag, (sieve analysis), and wood pellet fly ash (laser diffraction particle size analyzer using Shimadzu SALD-2300).

**Figure 2:**
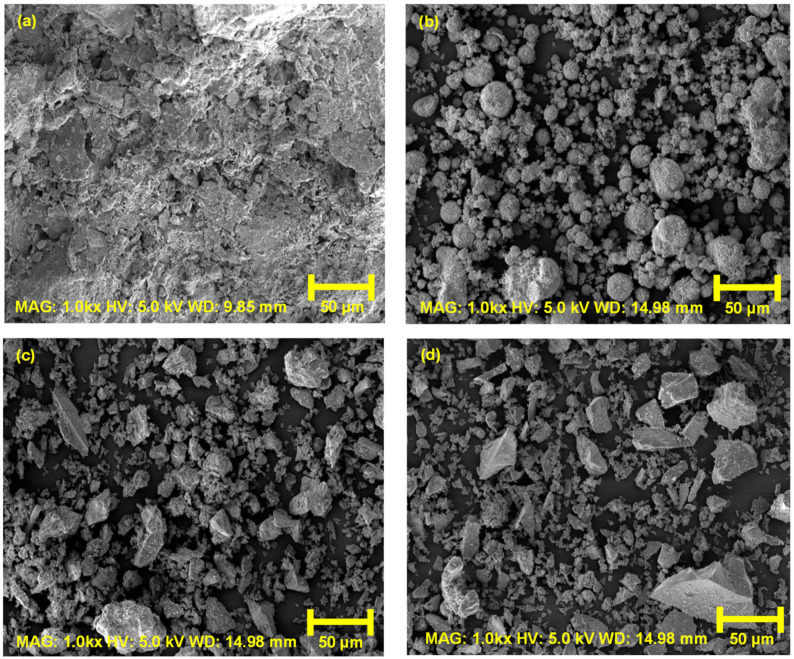
SEM images at 50 µm of (**a**) weathered granite soil, (**b**) wood pellet fly ash, (**c**) cement, and (**d**) ground granulated blast-furnace slag.

GGBS, on the other hand, is the by-product of processed iron from a blast furnace that can be used in the production of concrete in combination with commercial type 1 Portland cement. Figure 1 and Figure 2 show the particle size distribution (PSD) and SEM morphology of the materials used in this study. Table 1 presents the index properties of the host soil (e.g., minimum void ratio, e_min_; maximum void ratio, e_max_; specific gravity, G_s_, etc.) and binder constituents. The chemical components of the WABB constituents based on X-ray fluorescence (XRF) tests are shown in Table 2. All the components of WABB contain high calcium oxide (CaO) and silicon dioxide (SiO_2_), while GGBS has the highest aluminum oxide (Al_2_O_3_) content, all of which point to the possibility that it could react with active materials and form cementitious products. Notably, WA contains plenty of K, Na, and Ca, which are beneficial in supplying alkali ions to stimulate the latent hydration of GGBS and cement hydration. The WABB design mix was determined based on the best-performing cement mortar in terms of strength, as given in a previous study by Kim et al. [26,34,35,36]. 

**Figure 3:**
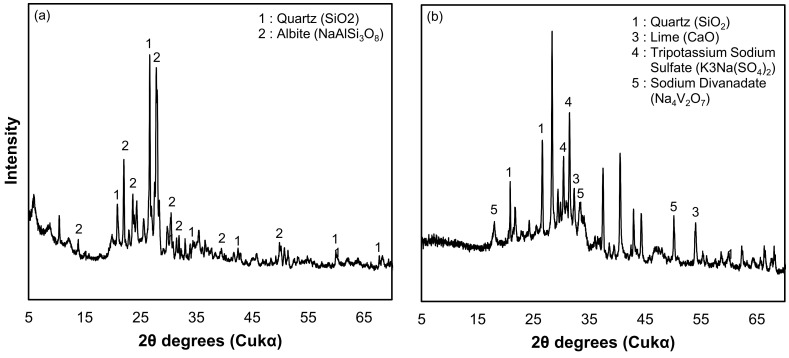

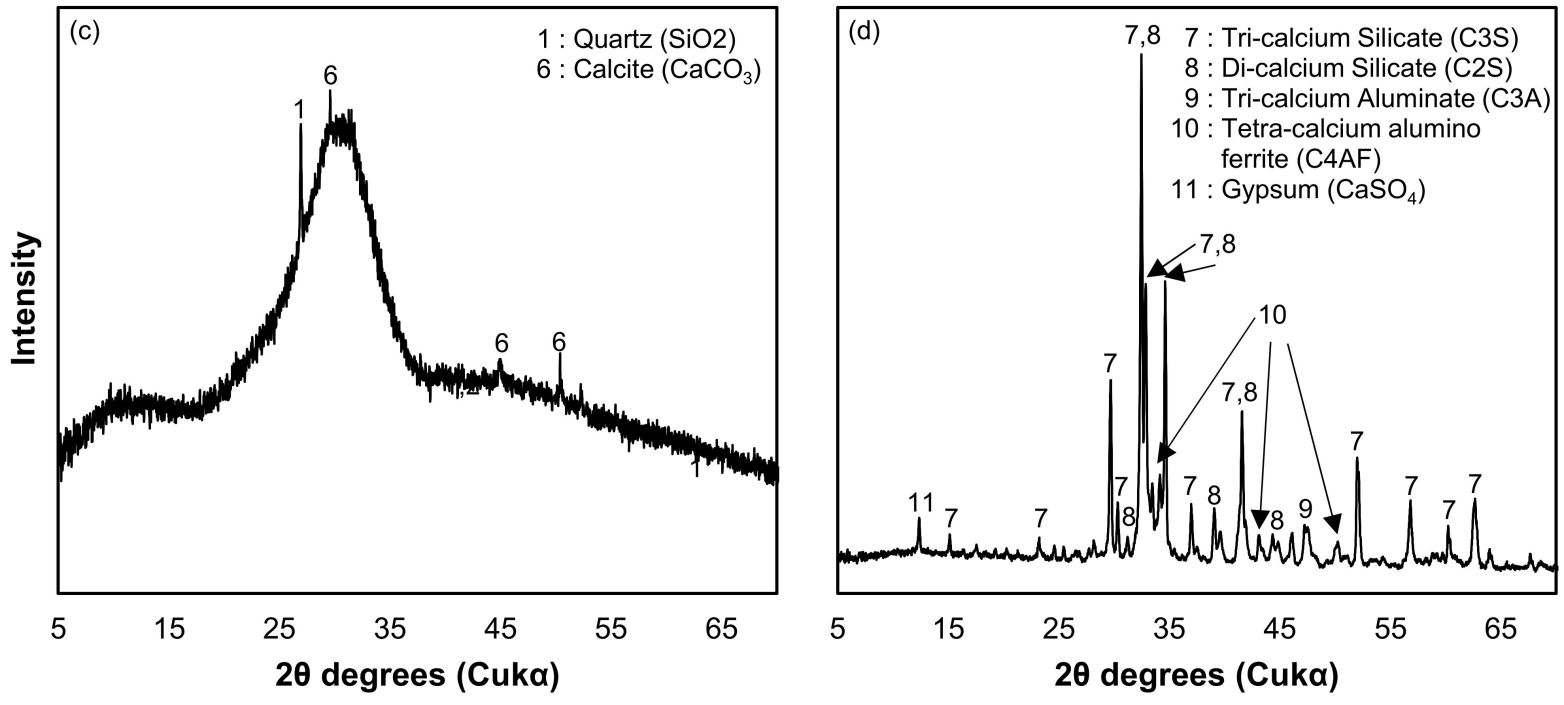
X-ray diffraction patterns of (**a**) weathered granite soil, (**b**) wood pellet fly ash, (**c**) GGBS, and (**d**) cement.

The phase diffractions of the individual constituents of WS and WABB were analyzed using XRD and are presented in Figure 3. The XRD reveals the WS silica (SiO_2_) and alumina (Al_2_O_3_) components through quartz and albite, respectively. The mineralogical constituents of WA are quartz (SiO_2_), lime (CaO), tripotassium sodium sulfate (K_3_Na(SO_4_)_2_), and sodium divanadate (Na_4_V_2_O_7_). The presence of lime in WA suggests its binding potential [15,23,36]. GGBS contains calcite (CaCO_3_), while cement is composed of tri-calcium silicate (C_3_S), di-calcium silicate (C_2_S), tri-calcium aluminate (C_3_A), tetra-calcium aluminoferrite (C_4_AF), and gypsum (CaSO_4_). The presence of gypsum helps to increase the soil strength [56]. The quartz minerals in WS, WA, and GGBS can enhance the Si-O-Si bonds, leading to a strength increase in stabilized soil [57].

Initially, modified proctor tests [58] were performed on weathered granite soil combined with wood pellet fly ash at 5%, 15%, and 25% by adding water to the dry host soil and mixing for two minutes to meet the optimum moisture content (OMC) and maximum dry density (MDD) in a separate project. As a result, the measured MDD and OMC for 5% WA were 1.932 g/cm^3^ and 12.0%; those for 15% WA were 1.917 g/cm^3^ and 10.5%; and those for 25% WA were 1.901 g/cm^3^ and 9.6.% [39]. These results indicate a minimal shift in OMC when combining WA and WS. Following that, the present study adapted the OMC and MDD for the WABB-stabilized weathered granite soil (WABB-WS) mix and employed dry mixing methods for the stabilized soil [59,60] to investigate the behavior change in compacted, weathered granite soil [61]. 

**Figure 4:**
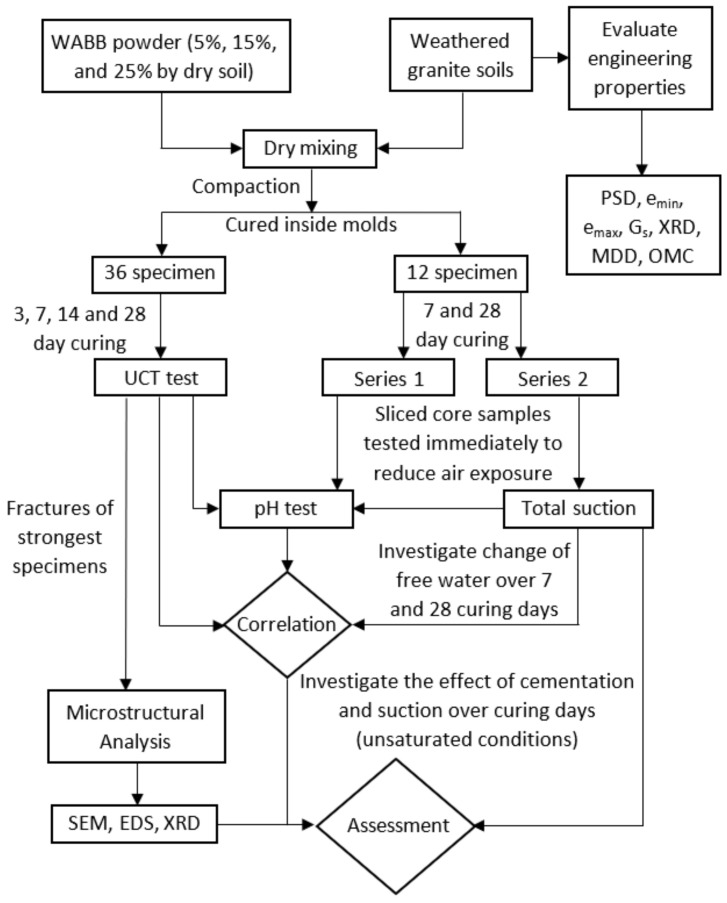
Flow chart of the testing plan for investigating the mechanical properties and microstructures of wood pellet fly ash blended binder-stabilized weathered granite soil.

Figure 4 illustrates the present study’s mixing condition and testing flow, including mechanical property and microstructure tests on 5%, 15%, and 25% of the WABB–WS specimens. In particular, the host soil was first sieved to eliminate coarse particles and then dried in an oven to make it ready for the experiments. Following the previously determined OMC, the dry soil was mixed with water and then with dry binders using a laboratory mixer for ten minutes until it became uniform. An 8 mm rod was used to tamp five layers of the soil–binder mixture into a cylindrical split mold, eventually aiming for a constant molding dry density of 95% relative compaction of 50 mm diameter and 100 mm high specimens. The specimens were finally prepared at a target dry density (*ρ_d_*) of 1.827 to 1.877 g/cm^3^, 1.812 to 1.858 g/cm^3^, and 1.796 to 1.834 g/cm^3^ for the 5%, 15%, and 25% WABB mixtures, respectively. To prevent the specimens from drying out, the specimen molds were sealed with plastic and cured at room temperature (25 ± 1 °C) in a closed tank partially filled with water (without direct contact with the water). The curing chamber humidity was 85 ± 2% and was monitored using a humidity meter. After 3, 7, 14, and 28 days, the specimens were extracted from the molds and examined. Dry curing was used to simulate stabilized WS projects installed near the ground surface and above the water table with no access to more water. Table 3 summarizes the testing type, mix proportions, and curing days for the WABB–WS specimens.

Unconfined compressive tests (UCT) [40] were performed to evaluate the unconfined compressive strength (*q_u_*) and the secant modulus (*E*_50_). The specimens were vertically and uniaxially loaded at 1%/min. After UCT tests, the pH of the mixed soil (approximately 9 mm and below) with the WABB specimens was monitored at 3, 7, 14, and 28 days (the specimens are marked with UCS and pH in Table 3) using a pH meter [62]. Subsequently, microstructural analyses were conducted through a series of SEM-EDS and XRD tests. In particular, another set of fractures (after UCT tests at 28 days) was reduced to small pieces to fit the mold size for the SEM-EDS tests (Model MIRA LMH) operated at 5 to 20 kV and a resolution of 1 nm. Also, XRD qualitative analysis (Model MiniFlex600-Rigaku) was conducted by investigating the reflected peaks and their varying intensities, which were operated at 40 kV and 30 mA on a Cu target (clear peaks at the 2θ region of 0 to 5 were not observed, thus the 2θ range of 5 to 70 is presented). A semi-quantitative analysis was conducted by measuring the intensity counts on the detected minerals [63] to evaluate the hydration of WABB at increasing dosage rates. These tests were conducted to validate the WABB’s contribution to the morphology of WS through the presence of cementitious materials relative to the mineral and chemical change over 28 curing days (see specimens marked under the MA column in Table 3). Moreover, twelve additional WABB–WS specimens were prepared and cured for 7 and 14 days and used for two testing series of pH and total suction tests (see specimens marked under the ST and pH columns in Table 3). These specimens were immediately extracted from the molds after the prescribed curing days to reduce air exposure. Then, the central cores of the specimens were divided into three parts consisting of the top, middle, and bottom portions. For Series 1, the core samples were carefully sliced into smaller intact specimens (approximately 9 mm and below). The specimens were immediately collected to maintain the initial conditions of the material, and the aluminum caps were filled to evaluate the total suction using a potentiometer (WP4C, METER Group) and the water content [64,65,66,67]. The use of WP4C for rapid total suction monitoring was effectively carried out to evaluate the hydraulic behavior of the unsaturated soils [68,69] and stabilized soils [65,70]. Furthermore, Series 2 testing was conducted by collecting the remaining intact samples and using them for pH tests. 

## 5. Results

### 5.1. Unconfined Compressive Strength and Secant Modulus

**Figure 5:**
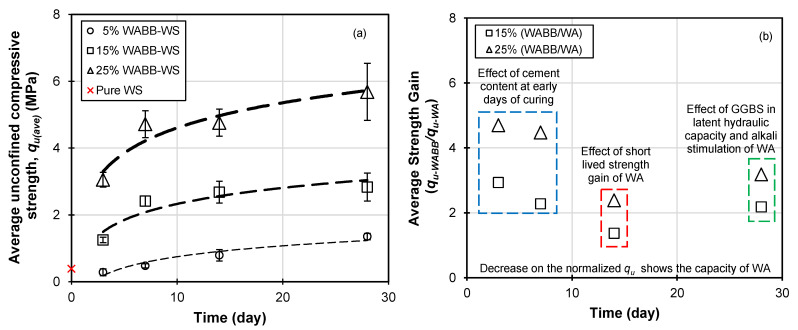
The effect of stabilization of WABB on (**a**) 3-to-28-day average *q_u_* of stabilized weathered granite soil and (**b**) normalized average *q_u_* of WABB–WS with Balagosa et al.’s [28] average *q_u_* of wood pellet fly ash-stabilized WS over 28 days (at 15% and 25% dosage).

Figure 5a presents the average unconfined compressive strength (*q_u(ave)_*) of the WABB-stabilized soil. The *q_u(ave)_* increases as the WABB dosage rate and curing days increase. In particular, the *q_u(ave)_* of the 5% WABB-treated specimens achieved 0.28 to 1.35 MPa from 3 to 28 days. The 3-day *q_u(ave)_* of the 5% WABB-treated specimens were almost identical, with the *q_u_* of the compacted WS exhibiting premature cementation at an early curing stage. The 15% WABB-treated specimen showed a higher *q_u(ave)_* of 1.25 to 2.83 MPa, whereas the 25% WABB–WS-treated specimen presented the highest strength, ranging from 3.05 to 5.68 MPa. All the WABB–WS-treated specimen *q_u(ave)_* values were 1.88 to 11.77 times higher than those of the compacted weathered granite soil. Figure 5b presents the normalized average strength gain of WABB–WS compared with pure wood pellet fly ash-treated WS (*q_u,WABB_/q_u,WA_*) to evaluate the effective strength gain of the wood pellet fly ash blended binder compared with pure ash over 28 days. The pure ash results were obtained from a previous study [39]. The results show that the strength gains for the 15% WABB range from 2.93 to 2.18, while those for the 25% WABB are from 4.70 to 3.18. These findings suggest that the cement content accelerates the hydration, while WA stimulates the alkali constituents in the WABB–WS matrix, leading to a high strength gain as early as days 3 and 7. Interestingly, a decrease in the ratio *q_u,WABB_/q_u,WA_* at 14 days was observed due to a significant increase in the denominator of the pure wood ash mixture strength. These findings imply the rapid consumption of lime content in biomass ash-stabilized soils, leading to a significant increase in 14-day strength and then a subsequent decrease for 28 days; this unique biomass ash stabilizing effect is the so-called short-lived strength gain [18,39]. On the other hand, the 28-day normalized strength gain was observed to increase due to the contribution of GGBS in the latent hydraulic capacity (strength gain with the increase in curing days) and the alkali stimulation of WA by the WABB’s components and the WS.

**Figure 6:**
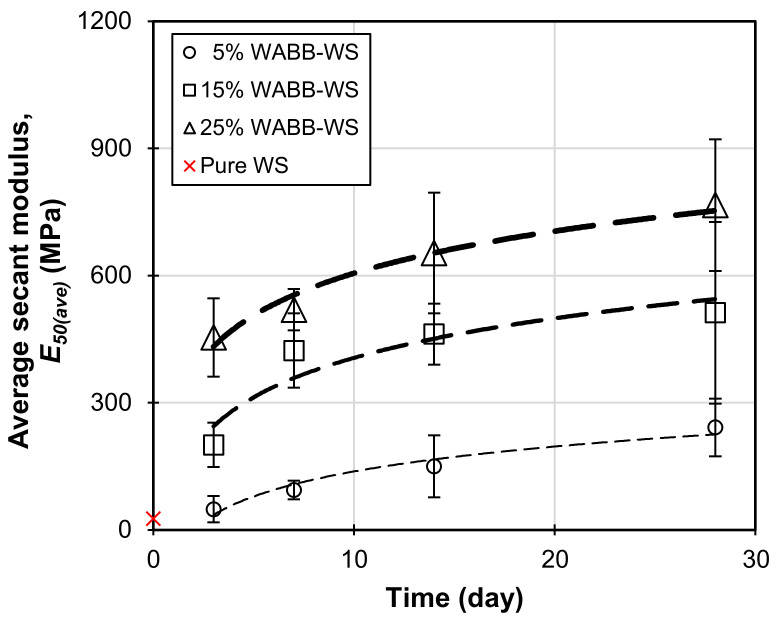
Average secant modulus of WABB-stabilized weathered granite soil.

Figure 6 portrays the average secant modulus (*E*_50*(ave)*_) of the WABB-treated WS over 28 curing days. The increase in the WABB dosage results in an increase in the *E*_50*(ave)*_ which is analogous to that observed in the *q_u(ave)_* profile. The lowest *E*_50*(ave)*_ among the mixtures was attained by the 5% WABB specimens, ranging from 48.70 to 241.55 MPa. The 15% WABB-treated specimens exhibited greater *E*_50*(ave)*_ values, ranging from 200.56 to 512.22 MPa, while the 25% WABB *E*_50*(ave)*_ ranged from 454.1 to 766.16 MPa up to 28 days. Interestingly, the 15% and 25% WABB contents exhibited accelerated cementation due to a high strength gain as early as days 3 and 7 of curing. These strength gains can be attributed to a higher proportion of cement (the primary contributor during the hydration process) [71,72,73,74] and GGBS (the latent hydration material) [54,75,76] and the WABB’s cation exchange reaction (which contains a considerable CaO content that may yield a potentially “lime-like” product) with WS (which contains soil silica SiO_2_ and alumina Al_2_O_3_), leading to the formation of cementitious minerals. The effect of WABB cementation on the WS microstructure is discussed in Section 5.2.

The slight increase in *q_u(ave)_* of the WABB-treated soil at 28 days implies latent hydraulic activity from GGBS and the low hydration capacity of WA [26,34,35,36,39]. Thus, the findings on strength and the moduli suggest the slow hydration of the WABB in the WS matrix and that the increase in strength will continue with extended curing days, which is beneficial for ground improvement projects. All the WABB–WS specimens satisfied the requirements for ground improvement projects, and the WS treated with the 15% WABB (cured 7 to 28 days) and 25% WABB (cured 3 to 28 days) met the sub-base strength requirement of 1.4 MPa for road structures of ACI-230 [77] and the Federal Highway Administration (FHWA) design manual deep mixing application criteria of 2.1 MPa for embankments and foundations [78]. On top of that, the 25% WABB–WS (cured 3 to 28 days) met the strength requirement of 3.5 MPa for base materials in road structures [77] and satisfied the design strength requirement of 4 MPa for the liquefaction mitigation of ground improvement projects [78]. 

The mechanical properties of soils treated with cementitious binders are affected by the unsaturated conditions and the development of the binder during the early stages of curing combined with the long-term conditions. During binder hydration, the soil pore structure experiences shrinkage due to the binder’s chemical stabilization, in which the larger pore of the soil structure containing water is emptied first, resulting in a reduction in the water meniscus and concurrent effects on suction [79]. Table 4 shows the change in water content (*w_c_*) measured from the fractures of the specimen (from UCT tests) after 3, 7, 14, and 28 curing days. As the percentage of the WABB is increased and the curing days are increased, less moisture loss (associated with external drying) is measured. The measured 7-day water content *w_c(7)_* for the 15% WABB–WS decreased from 8.88% to 8.02%, while the 25% WABB–WS showed a decrease from 8.04% to 7.21%, exhibiting identical moisture loss. Both samples exhibited greater desaturation values of more than 2% *w_c_* from the molding *w_c(m)_*. These changes in *w_c_* are relative to the WABB hydration mechanism, wherein, as the binder content increases, the soil capillary pores’ alteration increases and further leads to the concurrent effects of the suction and cementation of the WABB–WS matrix. This gradual consumption of *w_c_* in the WABB–WS matrix supports Phan et al.’s [80] claims regarding the importance of assessing the change in water absorption and retention (especially in the case of lower water-to-cement ratios) relative to the effect of the binder hydration on the mechanical properties of cementitious soils. Furthermore, the effects of suction at early curing days for cement-treated materials and its direct association with the desaturation rate are examined in Section 5.4.

### 5.2. Microstructural Analysis 

**Figure 7:**
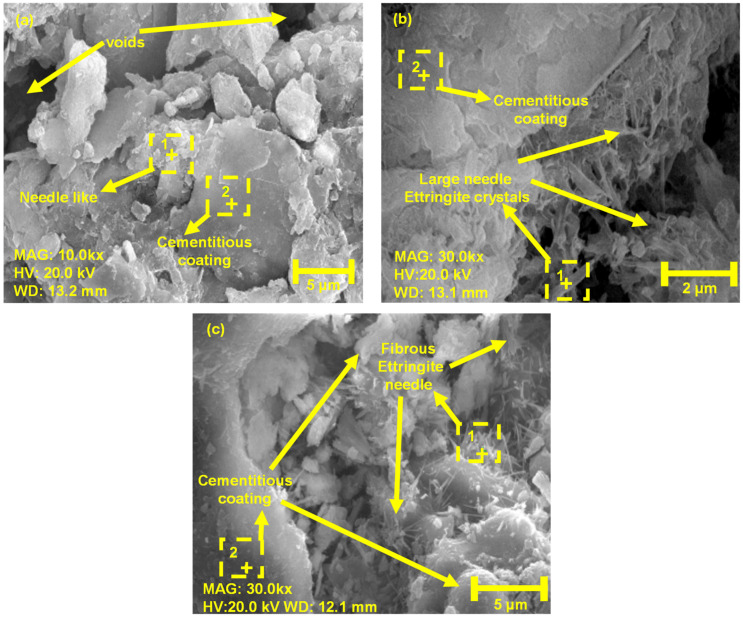
SEM images of compacted, treated weathered soils: (**a**) WABB5-C28-1 at 5 µm; (**b**) WABB15-C28-2 at 2 µm; (**c**) WABB25-C28-1 at 5µm (the selected areas were examined for elemental EDS analysis).

The soil morphologies after 28 days of WABB treatment are shown in Figure 7. In particular, Figure 7a,b show the SEM image of the microstructure of WABB5-C28-1 and WABB15-C28-2, which revealed visible needle-shaped ettringite minerals [81] and a small lamellar gel coating that is likely C-S-H and calcium aluminum silicate hydrate (C-A-S-H) minerals surrounding the WS particles. A few gelling products were observed, and apparent ettringite formations on the 5% and 15% WABB–WS may have caused the slower strength gain due to their filling in of pore spaces with dispersed porous structure (or visible holes) in the WS structure, resulting in almost equal 14- and 28-day *q_u_*. Figure 7c shows the increasing quantity of the gel-like coating of C-S-H and C-A-S-H minerals with a concurrent size reduction in ettringites when the 25% WABB (WABB25-C28-1) is introduced to WS; this coating uniquely appears in the 25% WABB specimen rather than in the lower dosage cases due to its higher cement proportion.

**Figure 8:**
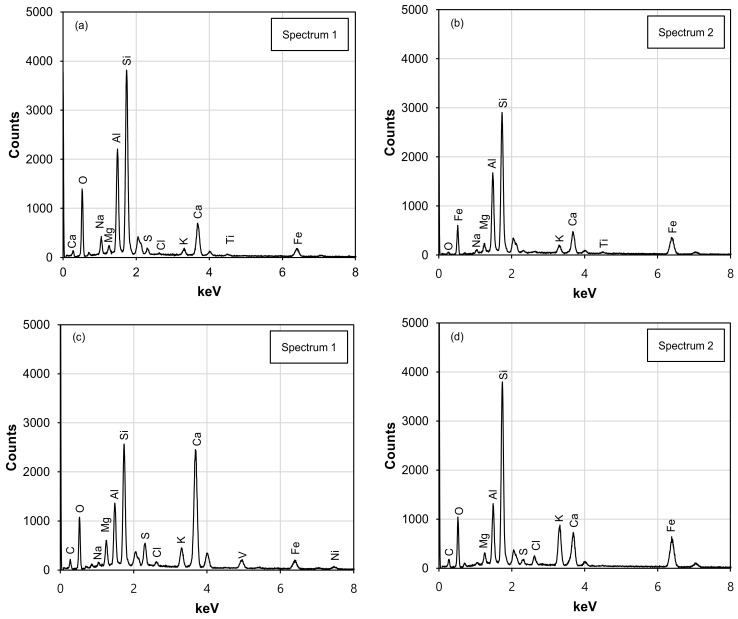

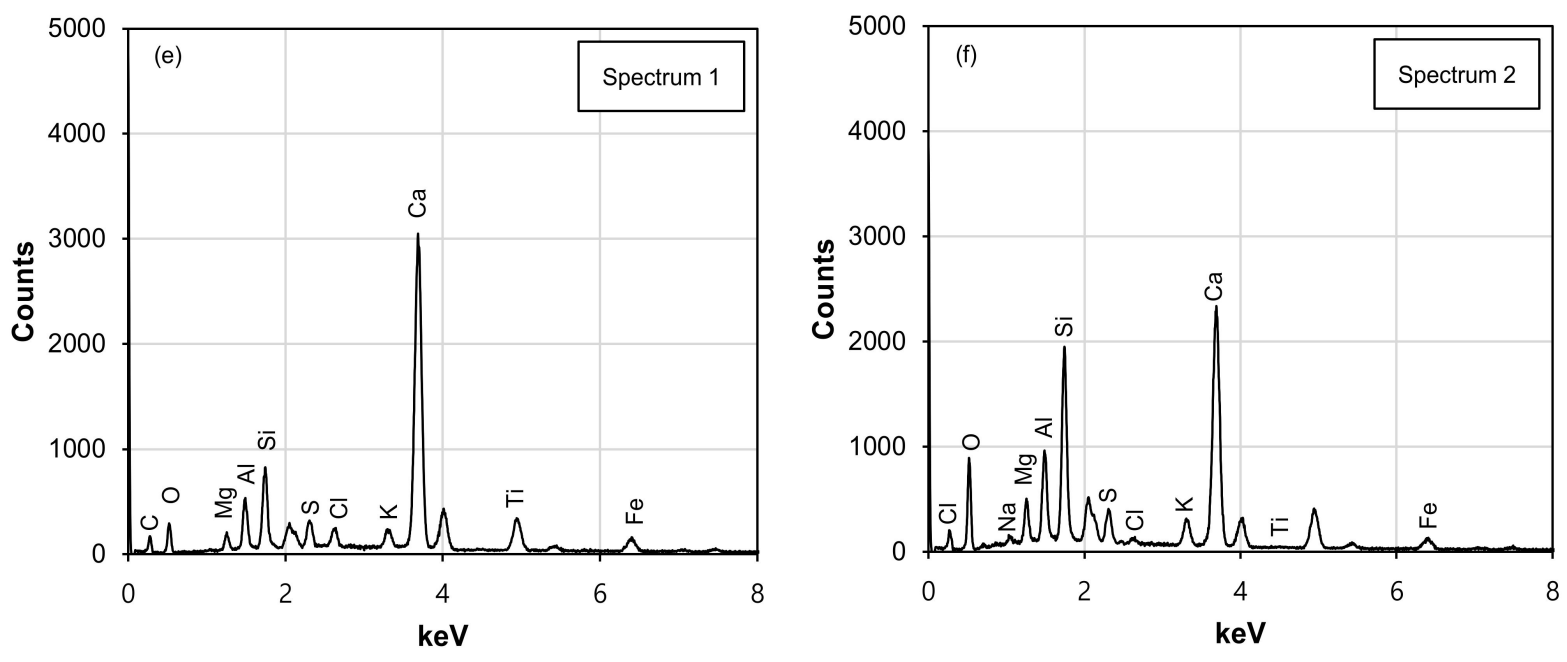
EDS diagrams of (**a**) Spectrum 1 and (**b**) Spectrum 2 in Figure 7a for WABB5-C28-1; (**c**) Spectrum 1 and (**d**) Spectrum 2 in Figure 7b for WABB15-C28-2; and (**e**) Spectrum 1 and (**f**) Spectrum 2 in Figure 7c for WABB25-C28-1.

Figure 8 and Table 5 show the EDS analysis on the square spectrum and the weight percentages of all the major chemicals present in Figure 7a–c (SEM images with dotted lines), respectively. As the WABB dosage rate increases, the calcium (Ca) peak intensities increase while the sodium (Na) and aluminum (Al) decrease, indicating WS mineralization. In particular, Figure 8a,c,e exhibit the major chemical components in Spectrum 1 of 5% WABB and 15% and 25% WABB–WS, respectively, which present significant calcium (Ca), silicon (Si), and oxygen (O) peaks (for Spectrum 1). It is therefore classified as C-S-H or C-A-S-H gel. Figure 8b,d,f show Spectrum 2, which has significant peaks in calcium (Ca), sulfur (S), aluminum (Al), and oxygen (O); thus, it is suspected to be ettringite gel. On the other hand, the decrease in Al as the WABB dosage increases suggests its participation in C-S-H development and its propensity to crystallize further into C-A-S-H gel structures over the curing days [2,82]. C-S-H, C-A-S-H, and ettringite gels are cementitious minerals associated with a strength increase in the soil matrix in ground improvement projects [83,84]. Their development depends on the soil type, water–binder ratio, cement content [85,86], and the unique chemical composition of alternative binders (i.e., silica-based or calcium-based biomass ash, fly ash, GGBS, etc.) that promote cementation [87,88]. Furthermore, the EDS analysis also detected K, Fe, Na, and Mg.

**Figure 9:**
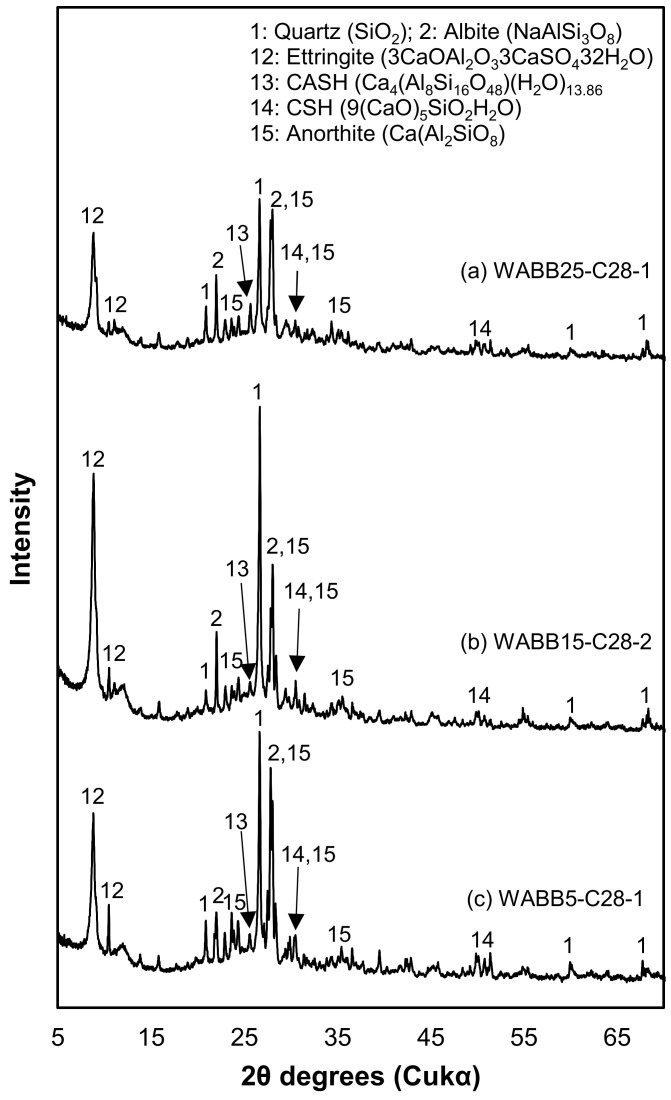
XRD diffraction patterns at 28 days: (**a**) WABB25-C28-1; (**b**) WABB15-C28-2; and (**c**) WABB5-C28-1.

Figure 9 presents the XRD results of WABB–WS at 28 days of curing. It can be observed that a decrease in mineral peaks in the common minerals in the WS, such as quartz and albite, is detected as the WABB dosage increases. In addition, new hydration products, such as ettringite, C-A-S-H, C-S-H, and anorthite minerals, were also detected. The presence of anorthite in all the XRD diffractograms of all the specimens indicated the occurrence of the reaction that resulted in C-S-H with a tendency for the infusion of C-A-S-H gel [89]. The production of anorthite in the present study supports and agrees with the previous cases in which biomass ash was discharged from palm oil fuel ash using commercially available precursors (e.g., fly ash, bottom ash, and GGBS) for the development of a new cementitious mortar [90]. These anorthite minerals also contributed to the strength of biomass ash blended with cement and GGBS in stabilized clay for road subgrade materials (consisting of natural gravel and sand with small amounts of clay) [54]. On the other hand, Ca(OH)_2_ was not detected through XRD analysis. This might be attributed to OH- (in a typical Ca(OH)_2_ during cement hydration), which actively disintegrates the Ca-O, Al-O, and Si-O of GGBS and further contributes to the formation of hydration products [91]. However, it is postulated that Ca(OH)_2_ is also consumed as an alkali agent for GGBS together with alkali agents K^+^ and Na^+^ supplied from wood pellet fly ash during the WABB’s hydration process. Interestingly, the present study confirms the contribution of albite minerals in granite constituents when combined with cementitious binders in promoting C-S-H gel formations [39,90,92]. BFA with a smaller particle size promotes an increase in reactivity due to its larger surface area (measured using the Blaine method), facilitating the progression of hydration reactions through heterogeneous nucleation of hydration products [93,94], thus leading to enhanced stiffness of the soil matrix. Hence, the WA’s particle size may also contribute to the densification of WABB–WS specimens. It is noteworthy that the WA used in this study did not undergo any pre-processing treatments (i.e., grinding). Thus, the characteristic small particle of WA (as shown in Figure 1) may be utilized as a potential filler that fills the voids when mixed with coarse-sized materials [95,96]. The alteration of the granulometry of cementitious binders may affect the strength gain of stabilized soils [13,97]. This study aims to develop a binder with wood pellet fly ash and to improve the economic aspects involved in pre-processing treatments. Thus, the original powder of wood pellet fly ash and commercially available GGBS and cement were used. This approach certainly suggests economic advantages through the minimization of the costs associated with material pre-processing.

Table 6 presents the semi-quantitative analysis of the XRD results of WABB–WS. This analysis utilizes and adopts the methodology proposed by Padilla-Encinas et al. [63] for examining the changes in intensity counts of detected minerals in relation to the hydration of the binder. The alterations in intensity counts suggest the impact of higher WABB dosage on the cementation mechanism. This effect is shown by the partial conversion of WS into cementitious properties, hence supporting the results obtained from SEM-EDS analysis. Also, these findings suggest that the increase in WABB treatment on WS exhibits a continuous coating of C-S-H or C-A-S-H gels on the WS particles, with the 25% WABB identified as the optimal dosage rate contributing to an almost 15-fold increase in 28-day *q_u_* compared with compacted WS without binders (as shown in Figure 5a). The qualitative analysis and semi-quantitative analysis of the change in intensity counts suggest the preliminary development and conversion of albite-based weathered granite soil. The use of the Rietveld quantitative distribution analysis would have provided additional evidence on cementitious minerals [98]; however, it was not considered in this study due to the limitations of the institution’s facilities. Further investigation is required to address this aspect.

### 5.3. pH Tests

The pH values increased with the increase in WABB concentrations in the stabilized mixture. After 3 to 28 days of curing, the pH of the 5% WABB samples ranged from 11.88 to 11.44. A higher pH, between 12.22 and 11.85, was measured in the 15% WABB–WS specimen. The 25% WABB content exhibited the highest pH among the WABB–WS specimens, ranging from 12.65 to 12.05 over 28 curing days. The highest measured pH of 25% WABB–WS might be associated with the high gypsum content in cement, which helps expedite the early strength gain [91] at higher pH values, leading to the formation of extensive interlocking crystal structures within the soil matrix [99,100]. The decrease in pH content that was seen in all mixes may have been caused by the rapid consumption of lime-like minerals during the early curing phase (the first two weeks) and a concurrent reduction in the number of soil–lime reactions, which were later transformed into a new cementitious mineral. A pH value greater than 10 suggests that the amorphous SiO_2_ becomes more soluble [101], which aids the dormant hydraulic binding processes with GGBS, releasing SiO_2_ and Al_2_O_3_ to form a high-pH matrix [102]. 

### 5.4. Total Suction Measurement

**Figure 10:**
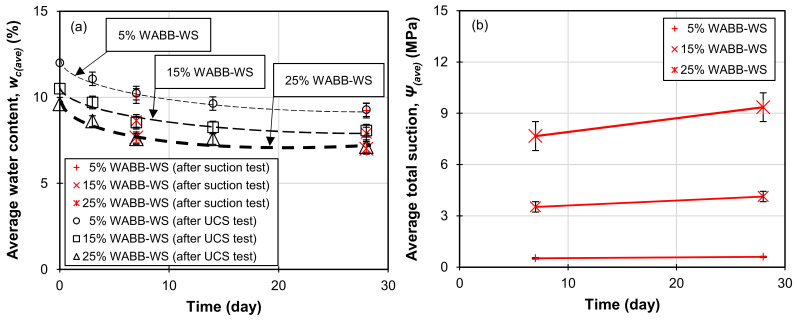
The effects of increasing WABB concentrations and curing days on (**a**) measured average water content following UCS and suction tests and (**b**) average total suction.

The WABB–WS mixtures used in this research were subjected to unsaturated conditions, resulting in decreased pore water and increased suction between grains. Therefore, monitoring any variations in suction (related to the change in water content) that occurred in the specimens over the curing period is essential. Figure 10 presents the alteration in the measured average water content (*w_c(ave)_*) and average total suction (*Ψ_(ave)_*) with the increase in the WABB dosage and curing days. All the specimens showed a decrease in *w_c(ave)_* due to the consumption of the free water of the WABB in the soil matrix during hydration, as shown in Figure 10a. This increase in desaturation was also observed with the higher WABB dosage due to the increasing quantity of cement and GGBS. Figure 10b illustrates the measured total suctions at 7 and 28 days. Increasing the WABB in the WS matrix increased *Ψ_(ave)_* during curing. After 7 and 28 days, the measured *Ψ_(ave)_* of the 5% WABB mixed specimen ranged from 0.51 to 0.60 MPa. For the 15% WABB sample, the *Ψ_(ave)_*ranged from 3.45 to 4.46 MPa. Subsequently, the 25% WABB–WS had the highest *Ψ_(ave)_* in the range of 7.46 to 9.42 MPa. As shown by the chemical stabilization caused by increasing the WABB in the WS, osmotic suction accounts for the majority of the measured increase in *Ψ_(ave)_*. These effects may be caused by the formation of new chemical bonds creating cementitious bridges between the WABB and WS particles due to the increasing ion concentration (as illustrated in Figure 7, Figure 8 and Figure 9). The contribution of the WABB’s suction and cementation to the strength and moduli of weathered granite soil over 28 curing days is discussed in Section 6.

## 6. Discussion: Cementation and Suction Development

**Figure 11:**
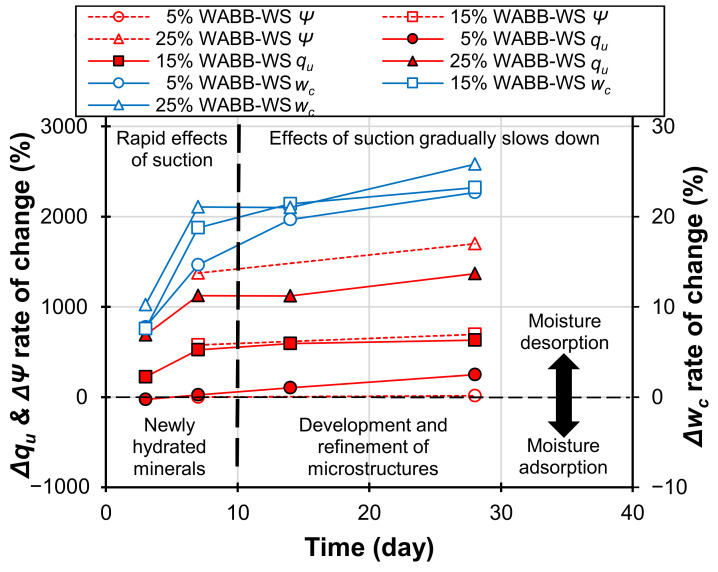
The effects of WABB’s hydration on moisture desorption and adsorption and its relative change on strength and total suction of WABB–WS specimens over curing days.

To analyze the WABB–WS strength-gaining mechanism provided between the particle cementation and the suction (for unsaturated conditions), the rate of change in *Ψ*, *q_u_*, and *w_c_* and its significance were investigated and defined as follows:

(1) $$∆\Psi \;\left(\%\right)=\left|\left(\frac{\Psi_{\left(i\right)}}{\Psi_{5\%WAB\left(7\right)}}-1\right)\times 100\right|$$

(2) $$∆q_{u}\;\left(\%\right)=\left|\left(\frac{q_{u(i)}}{q_{u\left(WS\right)}}-1\right)\times 100\right|$$

(3) $$∆w_{c}\;(\%)=\left|\left(\frac{w_{c(i)}}{w_{c(m)}}-1\right)\times 100\right|$$

where Δ*Ψ* = the rate of change in *Ψ*; *Ψ_(i)_* = the measured suction at a specified *ith* curing day; *Ψ_5%WABB(7)_* = the measured 7-day suction representing the early minimal suction contribution of 5% WABB–WS; *Δq_u_* = the rate of change in *q_u_*; *q_u(i)_* = the measured strength at a specified *i*^th^ curing day; *q_u(WS)_* = the measured 0-day strength of compacted weathered granite soil, representing the early strength of the pure soils under the minimal suction; *Δw_c_* = the rate of change in *w_c_*; *w_c(i)_* = the measured water content at specified curing days; and *w_c(m)_ =* molding water content. Figure 11 shows that as the WABB content and curing days increase, *Δq_u_* and *ΔΨ* increase while *Δw_c_* decreases. In addition, the observed rapid desaturation rate of all the WABB–WS specimens at 7 days agrees with and supports Abdul-Hussain and Fall [79] and Jing et al.’s [103] claim regarding the effect of cement (or cementitious material) hydration to suction on the early *q_u_* gains. Then, at 28 days, the measured relatively slower 28-day *Δw_c_* suggests the contribution of the WABB’s cementation capacity since more water is inscribed between the materialized cementitious minerals and the WS grains.

**Figure 12:**
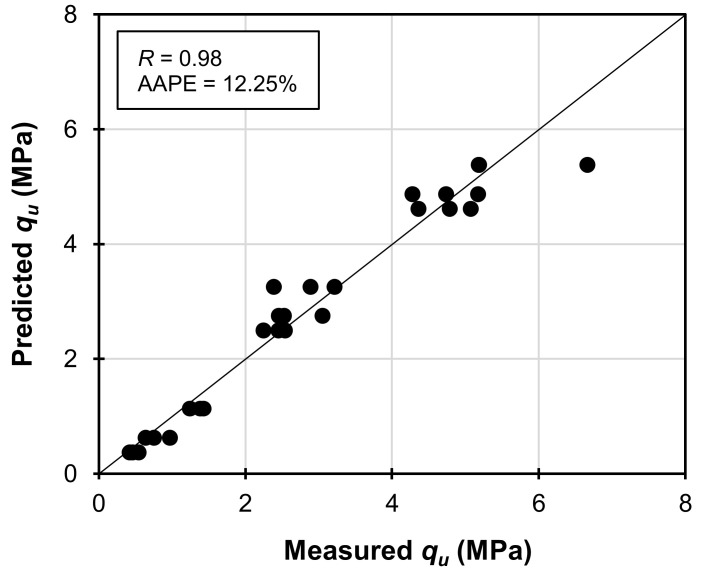
Comparison of the 7-to-28-day measured *q_u_* and predicted *q_u_* of WABB–WS specimens considering normalization from compacted weathered granite soil.

**Figure 13:**
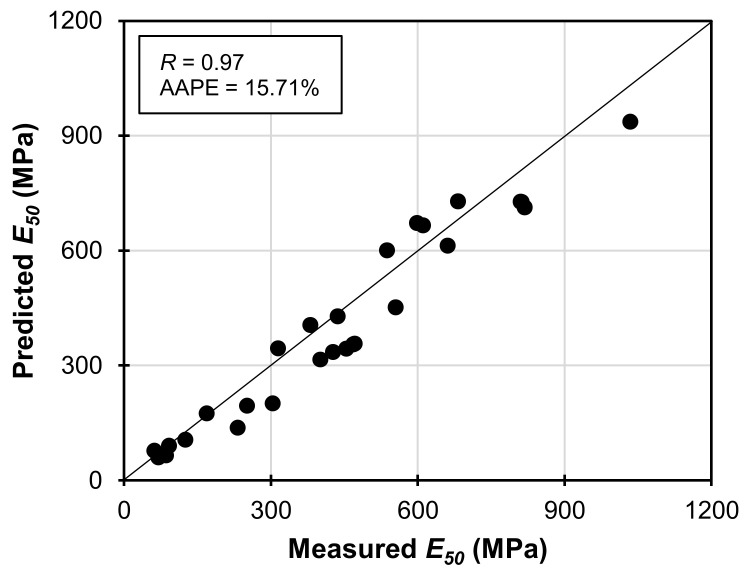
Comparison of the 7-to-28-day measured *E*_50_ and predicted *E*_50_ of WABB-WS specimens considering normalization from compacted weathered granite soil.

As the number of curing days is extended, the WABB material gradually develops a dense skeleton structure; thus, the majority of the strength improvement can be associated with the enhancement of the WS internal structure. However, due to the concurrent densification of the WABB–WS matrix and pore refinement, the formation of suction is still discernible [104]. Thus, the contribution of suction to the unsaturated WABB–WS strength gain mechanism slightly reduces. Despite the possibility of a suction-controlled strength gain, the results of this study agree with those of other cementitious soil studies, wherein the effect of suction on the strength gain of cementitious soils gradually decreases as the number of curing days increases [59,103,105,106]. The reduced contribution of suction is corroborated by the positive strength gain of the WABB–WS specimens due to the alteration in soil micropores through the newly developed cementitious minerals (i.e., C-S-H, C-A-S-H, Ettringite, and anorthite), leading to enhanced WS stiffness. Based on the aforementioned analysis, the Pearson correlation [107] was employed to assess the relative contribution of the 7-to-28-day unconfined compressive strength of WABB–WS (*q_u(_*_7_ *_to_*_28*)WABB-WS*_) with WABB cementation over the curing days. Then, a regression model was developed to predict the *q_u(_*_7_ *_to_*_28*)WABB-WS*_ using 70% of the total data as training data and 30% as cross-validation data. Lastly, the predicted and measured values were evaluated based on three factors: coefficient of determination (*R^2^*), correlation coefficient (*R*-value), and the average absolute percentage error (AAPE) [108,109], as follows:

(4) $$q_{u\left(7\;to\;28\right)WABB-WS}=212.24\times 10^{-3}\left(BDR\%\right)+36.33\times 10^{-3}\left(CD\right)-944.48\times 10^{-3}$$

where BDR = the ratio of dry wood pellet fly ash blended binder mass to dry soil mass in %; CD = curing days in the range of 7 to 28 days. The *q_u(_*_7_ *_to_*_28*)WABB-WS*_ had *R*-values of 0.18 and 0.96 for *BDR%* and *CD*, respectively. Figure 12 compares the values predicted by Equation (4) to the measured values, and this comparison yields an *R^2^* of 98.68, an *R*-value of 0.98, and an AAPE of 12.25%. These findings suggest that the predicted *q_u(_*_7_ *_to_*_28)*WABB-WS*_ using the Equation (4) model is in good agreement with the experimental results as displayed in the cross-plots. In addition, the *E*_50_ and *q_u_* of WABB-WS were normalized with the average *q_u_* and *E*_50_ of the compacted WS and correlated to derive a linear relationship between *E*_50_ and *q_u_* (*m* = 2.072; *n* = 27; *R^2^* = 95.27), as shown in Equation (5):

(5) $$E_{50\left(7\;to\;28\right)WABB-WS}=2.072\left(\frac{q_{u\left(7\;to\;28\right)WABB-WS}}{q_{u(WS-AVE)}}\right)\times E_{50(WS-AVE)}$$

where *E*_50*(*7_*_to_*_28*)WABB-WS*_ = the measured secant modulus of the WABB-stabilized WS from 7 to 28 days; *E*_50*(WS-AVE)*_ = the average secant modulus of the compacted WS without binders; *q_u(_*_7_ *_to_*_28*)WS-WAB*_ = the measured unconfined compressive strength of the WABB-stabilized WS at 7 to 28 days; and *q_u(WS-AVE)_* = the average unconfined compressive strength of the compacted WS without binders. Figure 13 presents the simulation of model Equation (5) with the experimental data. The obtained *R*-value of 0.97 and the AAPE of 15.71% indicate a good agreement between the predicted and measured values. Therefore, the *q_u_* and *E*_50_ of the compacted WS and *q_u(_*_7_ *_to_*_28*)WS-WABB*_ can be used to estimate the 7-to-28-day secant moduli of the WABB–WS mixtures. However, the prediction model Equations (4) and (5) are only applicable with the compacted WABB–WS prepared at a target dry density range of 1.796 g/cm^3^ to 1.877 g/cm^3^, a minimal range of *w_c(m)_* at 9.6% to 12.0%, and the testing conditions applied in the present study. Notably, the slower and premature strength gain of 5% WABB–WS and its microstructural developments further suggest that the optimal dosage rate for WABB applications in stabilizing WS should be 15 to 25% to achieve proper cementation. Nevertheless, the findings of the present study suggest the potential of the WABB in enhancing the stiffness of WS through its positive strength gain and the WABB’s cementation mechanism and in generating cementitious minerals with WS. These findings make the WABB utilization a cost-effective solution for developing BFA blended binders with a large volume of cement replacement [54,97,110] that can stabilize albite-based weathered granite soil.

## 7. Conclusions

An experimental investigation was conducted to validate the stabilizing mechanism of wood pellet fly ash blended binder to improve the mechanical properties and microstructures of naturally deposited weathered granite soil. The WABB was mixed with the WS at various proportions, and several series of laboratory tests were performed to evaluate the improvements in the soil matrix. This resulted in the following conclusions:

- This study provides the first observation of the clear cementation mechanism of WABB with natural soil, an albite-based weathered soil commonly found in inland areas, especially in Korea and Eastern Asia.
- The unconfined compressive strength (*q_u_*) and secant modulus (*E*_50_) increased with the increase in WABB content and curing days. These findings are attributed to the cement hydration and its influence on rapid early strength gain, GGBS latent hydraulic capacity, and the alkali stimulation of WA, leading to the combined hydration of the WABB with the WS SiO_2_ (through quartz) and Al_2_O_3_ (through albite) constituents.
- The hydration of the WABB in WS was verified to significantly alter the soil microstructure, as confirmed via XRD and SEM-EDS investigations. These analyses revealed the partially converted WS and the presence of newly formed cementitious minerals: ettringite, C-S-H, C-A-S-H, and anorthite.
- The strength development of the WABB–WS specimen is influenced by the total suction and cementation contributed by the increasing of the quantity of wood pellet fly ash blended binder at the early stage. After 7 days, the strength gain of WABB–WS was regulated with the WABB’s cementation, leading to a positive increase in the strength and moduli. These results corroborate the unique cementation effect of a three-component binder consisting of WABB with WS.
- Empirical equations were derived to estimate the mechanical properties *q_u(_*_7_ *_to_*_28*)*_ and *E*_50*(*7_ *_to_*_28*)*_ from the WABB dosage rate and curing days. The proposed model equation may have the potential to be applied in the design of the strength gain of WABB-stabilized weathered granite soil for ground improvement projects.

## Figures and Tables

**Table 1:** Properties of host soil and binders.

Properties	WS	WA	CEMENT	GGBS
Specific gravity, *G_s_*	2.67	2.31	3.15	2.85
Fine content passing #200 (%)	0.01	-	-	-
Maximum void ratio, *e_max_*	1.021	-	-	-
Minimum void ratio, *e_min_*	0.554	-	-	-
Grain size (mm)	*d_10_* = 0.447	*d_10_* = 2.636 × 10^−3^	*d_10_* = 0.362	*d_10_* = 0.354
	*d_50_* = 1.207	*d_50_* = 15.347 × 10^−3^	*d_50_* = 0.768	*d_50_* = 0.644
	*d_60_* = 1.490	*d_60_* = 20.233 × 10^−3^	*d_60_* = 0.951^3^	*d_60_* = 0.712
Soil classification, USCS	SP	-	-	-
Coefficient of uniformity, *c_u_*	3.333	-	-	-
Plastic index, PI (%)	NP	-	-	-
Optimum moisture content * (%)	11	-	-	-
Maximum dry density * (g/cm^3^)				
LOI		6.82	0.35	0.3
LSF	1.984	0	90.4	1

Note: * Modified proctor test (ASTM D1557-12e1); LOI: loss on ignition; LSF: lime saturation factor.

**Table 2:** Chemical composition of binders.

Components	WA (%)	Cement (%)	GGBS (%)
Na_2_O	3.47		
MgO	3.21	2.01	5.3
Al_2_O_3_	6.6	5.02	15.8
SiO_2_	23.25	21.88	33.4
P_2_O_5_	2.28		
SO_3_	8.4	1.83	0.3
Cl	2.8		
K_2_O	16.64	0.92	1.5
CaO	27.8	64.18	41.8
TiO_2_	0.43		
MnO	0.5		
Fe_2_O_3_	3.81	3.66	0.6
ZnO	0.14		
SiO	0.16		
etc.	0.50		

**Table 3:** Mix design and testing scheme.

Specimen ID	UCS	ST	pH	MA	Specimen ID	UCS	ST	pH	MA	Specimen ID	UCS	ST	pH	MA
WABB5-C3-1	✓				WABB15-C3-1	✓				WABB25-C3-1	✓			
WABB5-C3-2	✓				WABB15-C3-2	✓				WABB25-C3-2	✓			
WABB5-C3-3	✓		✓		WABB15-C3-3	✓		✓		WABB25-C3-3	✓		✓	
WABB5-C7-1	✓				WABB15-C7-1	✓				WABB25-C7-1	✓			
WABB5-C7-2	✓				WABB15-C7-2	✓				WABB25-C7-2	✓			
WABB5-C7-3	✓				WABB15-C7-3	✓				WABB25-C7-3	✓			
WABB5-C7-4		✓	✓		WABB15-C7-4		✓	✓		WABB25-C7-4		✓	✓	
WABB5-C14-1	✓				WABB15-C14-1	✓				WABB25-C14-1	✓			
WABB5-C14-2	✓				WABB15-C14-2	✓				WABB25-C14-2	✓			
WABB5-C14-3	✓		✓		WABB15-C14-3	✓		✓		WABB25-C14-3	✓		✓	
WABB5-C28-1	✓			✓	WABB15-C28-1	✓				WABB25-C28-1	✓			✓
WABB5-C28-2	✓				WABB15-C28-2	✓			✓	WABB25-C28-2	✓			
WABB5-C28-3	✓				WABB15-C28-3	✓				WABB25-C28-3	✓			
WABB5-C28-4		✓	✓		WABB15-C28-4		✓	✓		WABB25-C28-4		✓	✓	
WS-1	✓		✓	✓	WS-2	✓				WS-3	✓			

Note: WABB5 = specimen with 5% WABB (wood pellet fly ash blended binder); WABB15 = specimen with 15% WABB content; WABB25 = specimen with 25% WABB content; The WABB content is defined as dry mass ratio of WABB to WS; C3 = three curing days; C7 = seven curing days; C14 = fourteen curing days; C25 = twenty-five curing days. The last number in the specimen IDs is the testing sequence. ST = suction tests; MA = microstructural analysis using a series of XRD, SEM, and EDS.

**Table 4:** Measured water content after unconfined compressive tests.

Mixtures	w_c(m)_	3 Days	7 Days	14 Days	28 Days
WS + 5% WABB	12.00	11.07	10.24	9.64	9.28
WS + 15% WABB	10.50	9.70	8.53	8.25	8.06
WS + 25% WABB	9.60	8.62	7.58	7.57	7.12

Note: Measured *w_c_* is in % and average.

**Table 5:** Quantitative results of the EDS analysis on WABB-treated soils.

Element	5% WABB		15% WABB		25% WABB	
	S1 (%)	S2 (%)	S1 (%)	S2 (%)	S1 (%)	S2 (%)
Oxygen (O)	43.58	29.15	39.91	34.70	48.11	26.25
Aluminum (Al)	10.92	16.28	5.95	6.13	4.76	5.93
Sulfur (S)	0.75	-	2.17	0.56	1.58	1.16
Sodium (Na)	2.61	1.31	0.84	0.41	0.40	0.35
Silicon (Si)	18.53	25.89	9.29	16.73	7.68	18.86
Calcium (Ca)	9.03	7.36	22.85	7.68	29.02	38.73
Ca:Si	0.49	0.28	2.46	0.45	3.78	2.05
Al:Ca	1.21	2.21	0.26	0.79	0.16	0.15
Si:Al	1.70	1.59	1.56	2.73	1.61	3.18
Suspected minerals	C-S-H, C-A-S-H	Ett	C-S-H, C-A-S-H	Ett	C-S-H, C-A-S-H	Ett

Note: Results are from the weight content (%) from the EDS tests; S1 = spectrum of interest 1; S2 = spectrum of interest 2; Ett = ettringite; C-S-H = calcium silicate hydrates; C-A-S-H = calcium aluminosilicate hydrates.

**Table 6:** Semi-quantitative analysis based on the intensity peaks of WABB–WS specimens.

Minerals		5% WABB	15% WABB	25% WABB	Norm. 15% WABB	Norm. 25% WABB
	2θ (Cuka)	Int.	Int.	Int.	Norm. Int.	Norm. Int.
Quartz	20.71	7556.84	5206.40	6844.66	0.69	0.91
Quartz	26.50	30,171.54	39,293.70	19,697.65	1.30	0.65
Quartz	59.97	2519.38	2026.42	2051.53	0.80	0.81
Quartz	67.70	2693.04	3101.29	2873.15	1.15	1.07
Albite	21.81	8487.33	12,198.67	10,731.32	1.44	1.26
Anorthite	22.76	6061.73	5709.27	5404.96	0.94	0.89
Anorthite	36.47	4174.35	4481.96	5163.72	1.07	1.24
Ettringite	8.66	20,442.82	31,212.72	15,734.26	1.53	0.77
Ettringite	10.38	9438.60	7872.78	5018.26	0.83	0.53
C-A-S-H	25.44	5932.98	6710.39	7224.77	1.13	1.22
Albite, anorthite	27.76	25,880.79	20,197.73	18,570.71	0.78	0.72
C-S-H, anorthite	30.40	5738.28	6346.06	5223.20	1.11	0.91

Note: Int. = intensity; Norm. = normalized; and Norm. Int. = n intensity using the values of 5% WABB as the denominator.

## Data Availability

Some data are available from the corresponding author by request.
